# Overexpression of p62/IMP2 can Promote Cell Migration in Hepatocellular Carcinoma via Activation of the Wnt/β-Catenin Pathway

**DOI:** 10.3390/cancers12010007

**Published:** 2019-12-18

**Authors:** Mengtao Xing, Pei Li, Xiao Wang, Jitian Li, Jianxiang Shi, Jiejie Qin, Xiaojun Zhang, Yangcheng Ma, Giulio Francia, Jian-Ying Zhang

**Affiliations:** 1 Department of Biological Sciences & NIH-Sponsored Border Biomedical Research Center, The University of Texas at El Paso, El Paso, TX 79968, USA; mxing@miners.utep.edu (M.X.); pli2@utep.edu (P.L.); xwang6@utep.edu (X.W.); jli4@utep.edu (J.L.); jshi@utep.edu (J.S.); jqin@utep.edu (J.Q.); xzhang5@miners.utep.edu (X.Z.); yma3@miners.utep.edu (Y.M.); 2 Henan Medical and Pharmaceutical Institute, Zhengzhou University, Zhengzhou 450052, Henan, China

**Keywords:** p62/IMP2, hepatocellular carcinoma (HCC), metastasis, EMT, β-catenin

## Abstract

p62/IMP2 is an oncofetal protein that was first reported as a tumor-associated antigen in hepatocellular carcinoma (HCC). In our previous studies, we demonstrated a high frequency of p62/IMP2 autoantibodies appearing in various types of cancer. Therefore, we hypothesize that p62/IMP2 plays an important role in the progression of HCC, although the mechanism remains to be explored. In this study, we evaluated the expression of p62/IMP2 protein both in human tissues and liver cancer cell lines by immunohistochemistry and western blotting analysis and found that p62/IMP2 protein is overexpressed in human HCC tissue in comparison to normal human liver tissue. To explore the role that p62/IMP2 plays in HCC, p62/IMP2 was knocked out in two p62/IMP2-positive liver cancer cell lines (SNU449 and HepG2). Due to the low expression level of p62/IMP2 in SNU449, we overexpressed p62/IMP2 in this cell line. We subsequently demonstrated that high expression of p62/IMP2 in both cell lines can promote cell migration and invasion abilities in vitro by activating the Wnt/β-catenin pathway. We also used the Wnt/β-catenin pathway inhibitor, XAV 939, and a phosphoproteome assay to confirm our findings. *Conclusion:* Our results suggest that p62/IMP2 is an essential regulator of Wnt signaling pathways and plays an important role in HCC progression and metastasis.

## 1. Introduction

Liver cancer was predicted to be the fourth leading cause of cancer deaths worldwide in 2018. HCC is the most common type of primary liver cancer [1]. Many studies indicate that HCC is caused by the accumulation of genomic mutations. In one human HCC nodule, the coding region within the genome was accumulated with an average of 40 functional somatic mutations [2]. Among them, the mutations in the β-catenin gene were shown to occur frequently in human HCC [3]. In addition, the Wnt/β-catenin pathway is frequently activated in patients with HCC, resulting in a more aggressive HCC subtype that carries a poor prognosis [4,5,6].

Insulin-like growth factor 2 mRNA-inding protein family members (IMP1, p62/IMP2, IMP3) are involved in embryonic development with a biphasic expression [7]. All the IMP family members share a highly conserved structure and have similar functions. Their characteristics include two N-terminal RNA recognition motifs (RRMs) and four heteronuclear ribonucleoproteins (hnRNPs) K-homology (KH) domains in their C-terminal regions [8]. IMPs are primarily cytoplasmic, and therefore bind with various mRNA to regulate their stability, translatability, and localization [9]. IMPs are considered oncofetal proteins, and their overexpression in various types of cancers has been reported [8,9].

p62/IMP2 was originally reported as a tumor-associated antigen (TAA) in HCC [10]. The high frequency of anti-p62/IMP2 autoantibody occurrence in the serum samples from various types of cancer patients implies that p62/IMP2 may play a role in cancer progression [11,12,13,14]. Several cancer-associated p62/IMP2 target mRNAs have been identified. They include CCN2 mRNA [15], IGF2 mRNA [7], and c-Myc mRNA [15]. Some studies have reported that p62/IMP2 is involved in carcinogenesis. When phosphorylated by mTOR, p62/IMP2 stabilizes HMGA1 and stimulates the expression of IGF2, potentially leading to increased cancer cell proliferation [16]. By activating the IGF2/PI3K/Akt pathway, p62/IMP2 can conceivably promote proliferation, migration, invasion, and epithelial to mesenchymal transition (EMT) of glioblastoma cells [17]. Recently, p62/IMP2 has been shown to enhance the migration ability of cancer cells and promote cancer metastasis. A cluster of p62/IMP2 targets (LIMS2, TRIM54, and LAMB2) are considered to be involved in the downstream effects of p62/IMP2 expression that indeed increased cell migration, cell adhesion, and cytoskeleton remodeling, which can promote the metastatic behavior of cancer cells [18]. A recent study from our group demonstrated that the overexpressed p62/IMP2 enhances the expression of β-catenin and CTGF, which therefore heightens cell migration and reduces cell adhesion in breast cancer cells [15]. Another group also reported that p62/IMP2 and IMP3 cooperate to downregulate the progesterone receptor and promote metastasis in triple-negative breast cancer cells [19]. p62/IMP2 can be considered as an indicator of Wnt/β-catenin signaling pathway in HCC, further triggering genomic instability and an invasive phenotype [4,20].

In the present study, we showed that the overexpression of p62/IMP2 is observed in HCC tissues and cell lines. By modulating the expression of p62/IMP2, we found that overexpression of p62/IMP2 significantly increases the migration in vitro ability of liver cancer cell lines. In addition, the expression of p62/IMP2 and β-catenin appear to be correlated. Our study suggests that p62/IMP2 may regulate the migration of liver cancer cells by activating the Wnt/β-catenin pathway.

## 2. Results

### 2.1. p62/IMP2 is Overexpressed in Human HCC Tissues and Cell Lines

**Figure 1:**
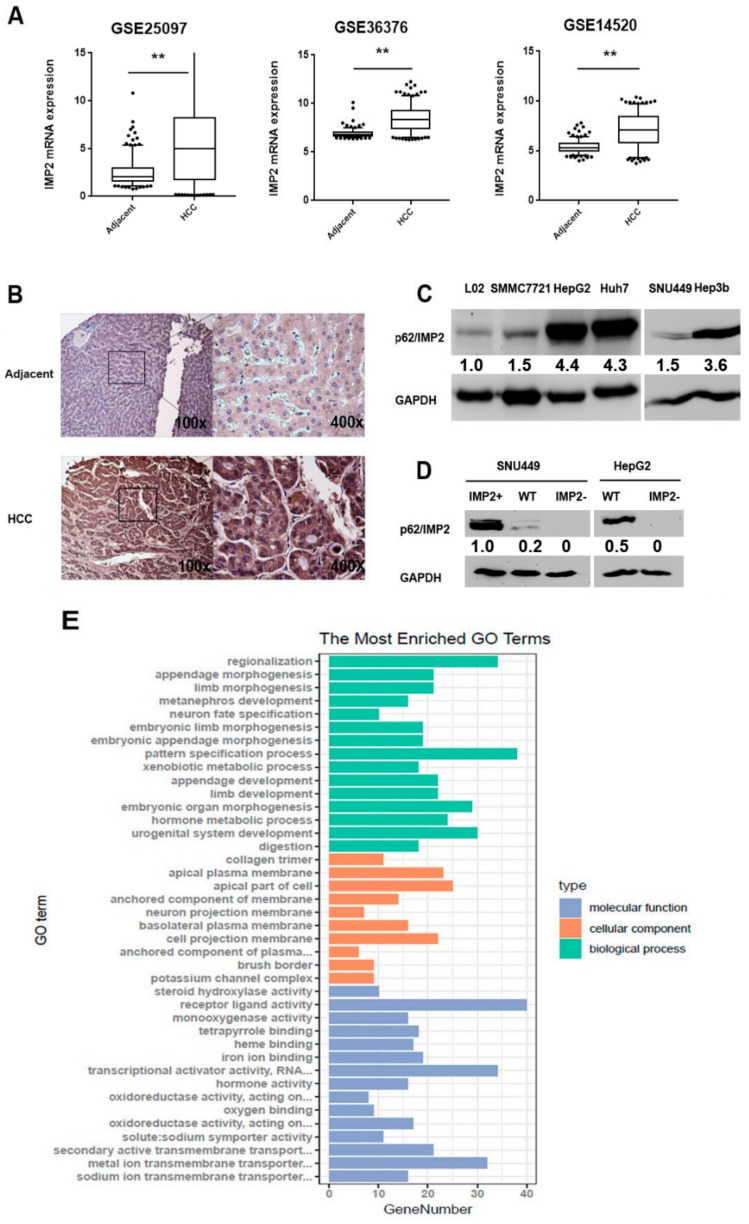
p62/IMP2 is overexpressed in HCC tissues and cell lines. (**A**) mRNA expression of p62/IMP2 in tumor tissues and their controls in datasets GSE 25097, GSE 36376, and GSE 14520, respectively. * *p* < 0.05, ** *p* < 0.01 (**B**) Immunohistochemical staining of p62 in liver cancer tissue and adjacent normal tissue slides. Weak stain pattern of p62 in representative adjacent normal tissue, and positive stain pattern of p62 in representative liver cancer tissue (×100 and ×400 magnification). (**C**) p62/IMP2 protein expression tested by western blotting analysis for the human fetal cell line L02 and five liver cancer cell lines. (**D**) p62/IMP2 overexpression (IMP2+) and knockout (IMP2-) in liver cancer cell lines were verified by western blotting analysis. (**E**) Gene ontology analysis was performed between the p62/IMP2 high expression group and low expression group in dataset GSE 14520.

We first investigated p62/IMP2 expression in a large cohort of HCC patients from the Gene Expression Omnibus (GEO) database (accession numbers GSE 25097, GSE 36376, GSE 14520). Compared with adjacent tissues of HCC, p62/IMP2 gene *(IGF2BP2)* expression in HCC tissues was significantly upregulated in all three datasets (Figure 1A). To examine the expression level of p62/IMP2 in HCC tissues, we performed immunohistochemistry (IHC) analysis on a tissue array, including 40 HCC tissues and 30 normal liver tissues. The expression of p62/IMP2 was scored by the immune-staining intensity and positive immune-staining cell area. p62/IMP2 protein was overexpressed in human HCC tissues compared with normal human liver tissues (score = 10.30, *n* = 40 vs. score = 5.23, *n* = 30, *p* < 0.05) (Table 1). However, the expression of p62/IMP2 was not significantly different between HCC tissues of different stages, which indicated that the overexpression of p62/IMP2 occurs in early HCC progression. The color scores between HCC tissues and normal tissues were significantly different while the area score results were similar. Representative examples of the weak p62/IMP2 stain pattern of adjacent normal tissue and strong p62/IMP2 stain pattern of HCC tissue are shown in Figure 1B. In addition, western blotting analysis was performed to examine p62/IMP2 protein expression in the non-tumorigenic liver cell line L02 and five liver cancer cell lines. p62/IMP2 were overexpressed in all liver cancer cell lines; in contrast, L02 cells showed a relatively low expression level of p62/IMP2. Interestingly, p62/IMP2 was highly overexpressed in well-differentiated cell lines (HepG2, Hep3B, and Huh7), whereas they were slightly overexpressed in poorly differentiated cell lines (SNU449) (Figure 1C) [21], which supported our IHC results that p62/IMP2 overexpression may occur in the early stage of HCC.

### 2.2. p62/IMP2 Does Not Significantly Promote Cell Proliferation but Reduces Cell Population Dependence and Enhances Colony Formation

**Figure 2:**
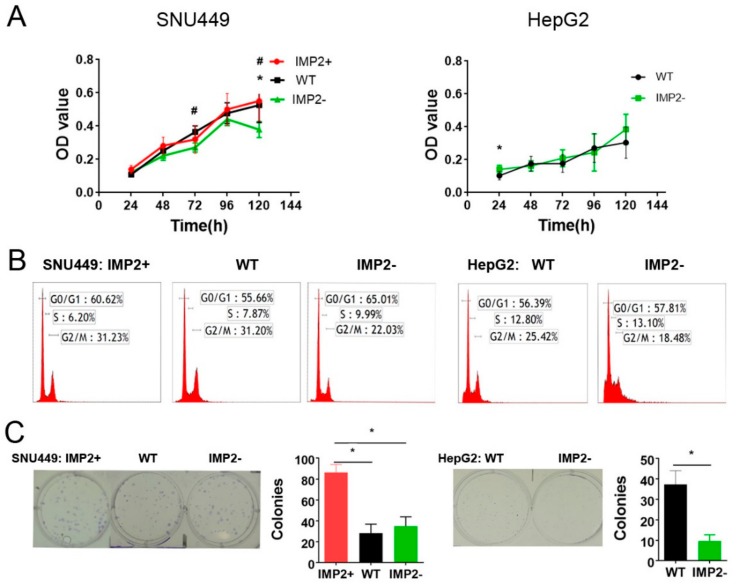
P62/IMP2 does not significantly promote cell proliferation but reduces cell population dependence and enhances the ability to form colonies (**A**) Growth curve of the cell proliferation assay in SNU449 and HepG2 cells. SNU449 IMP2+ vs. SNU449 IMP2−: # *p* < 0.05; SNU449 (WT) vs. SNU449(IMP−): * *p* < 0.05 (two-way ANOVA, *n* = 3). HepG2 (WT) vs. HepG2 (IMP−): * *p* < 0.05 (multiple *t*-test, *n* = 3). (**B**) Cell cycle of p62/IMP2 variants tested by flow cytometry. (**C**) Plate clonogenic assay p62/IMP2 variants from two liver cancer cell lines. High expression of p62/IMP2 could promote colony formation ability in liver cancer cells. * *p* < 0.05 (one-way ANOVA, *n* = 3).

To explore the biological roles of p62/IMP2 in HCC progression, p62/IMP2 was knocked out in a well-differentiated liver cancer cell line (HepG2) and in the poorly differentiated liver cancer cell line (SNU449). Furthermore, due to the low expression level of p62/IMP2 in SNU449, we performed a transfection experiment to overexpress p62/IMP2 in this cell line (Figure 1D). All the generated variants were tracked for 5 days in a proliferation assay to examine their relative growth rate. However, regardless of whether p62 was overexpressed or knocked out, we did not observe a consistent impact on the proliferation in either cell line (Figure 2A). The cell cycle distribution of each variant was measured by flow cytometry. The results support our proliferation assay results in that the cell cycles’ profiles were not significantly altered by the transfections. The only exception to this was that the cell ratio in the G2/M phase of IMP2 knockout cells decreased by 7% to 9%, compared with their corresponding wild-type parental cells (Figure 2B). Surprisingly, in a clonogenic assay, p62/IMP2-overexpressed cells showed a higher colony formation rate in both cell lines (Figure 2C). At the same time, the clone formation rate of SNU449 IMP2 knockout cells had a moderate increase as well. The clone formation rate reflects two important features: Cell population dependence and proliferation ability. Considering that overexpression or knocked out p62/IMP2 did not alter the proliferation ability in both liver cancer cell lines, our results indicated that the change of p62/IMP2 expression enhanced the ability of growing without population dependence.

### 2.3. Overexpression of p62/IMP2 Promotes Cell Migration in HCC

**Figure 3:**
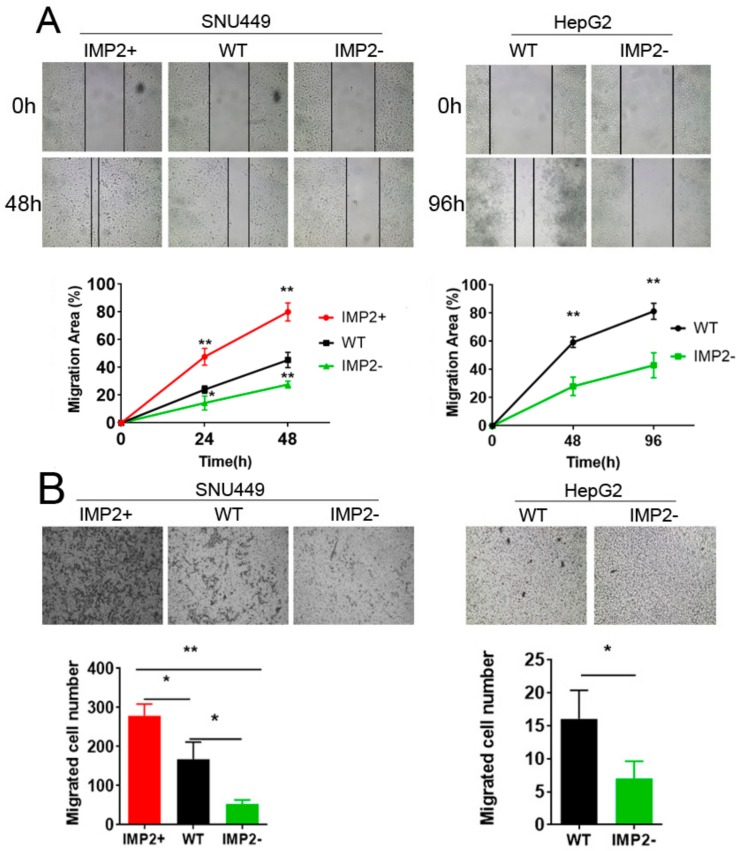
Overexpression of p62/IMP2 promotes cell migration in HCC. (**A**) The wound healing assay in p62/IMP2 variants from two liver cancer cell lines. * *p* < 0.05, ** *p* < 0.01, transfected cells compared with wild-type cells (two-way ANOVA and multiple *t*-test, *n* = 3). (**B**) The transwell migration assay in p62/IMP2 variants from two liver cancer cell lines. The image of transwell migration assay for p62/IMP2 variants from a random area under a 100× microscope. * *p* < 0.05, ** *p* < 0.01 (one-way ANOVA and *t*-test, *n* = 3). Values represent the mean ± SEM of three independent measurements.

We separated the tumor group of GSE 14520 into two groups based on the median *IGF2BP2* expression. In total, 900 differentially expressed genes (DEGs) were found between the IMP2 high expression group and IMP2 low expression group and used for a gene ontology (GO) analysis (Table S1). The cellular component analysis showed that most proteins expressed by DEGs are located in the collagen trimer, apical plasma membrane, apical part of the cell, and anchored component of the membrane, where the located proteins are highly related to the cell migration ability and EMT [22,23] (Figure 1E). In addition, the FunRich biological pathway and gene interaction analysis showed that part of the DEGs are related EMT and involved in the regulation of the Wnt/β-catenin pathway (Figures S1 and S2). These results might be indirect evidence and suggested that p62/IMP2 is involved in the regulation of cell migration. Therefore, we performed two in vitro assays, a wound healing assay and a transwell migration assay, to investigate the cell migration properties. Although the migration rate of hepG2 cells is significantly lower than SNU449 cells, both assays showed consistent results in that overexpressed p62/IMP2 can promote liver cancer cell migration in vitro. Overexpression of p62/IMP2 in liver cancer cells increased ‘wound’ closure by 40% to 50% compared to wild-type cells, which also significantly increased the number of transmembrane cells in the transwell migration assay (Figure 3A,B). Furthermore, a transwell invasion assay was done, which illustrated that overexpression of p62/IMP2 can augment the invasion ability of liver cancer cells (Figure S3).

### 2.4. Overexpression of p62/IMP2 Can Induce EMT through the Wnt/β-catenin Pathway 

**Figure 4:**
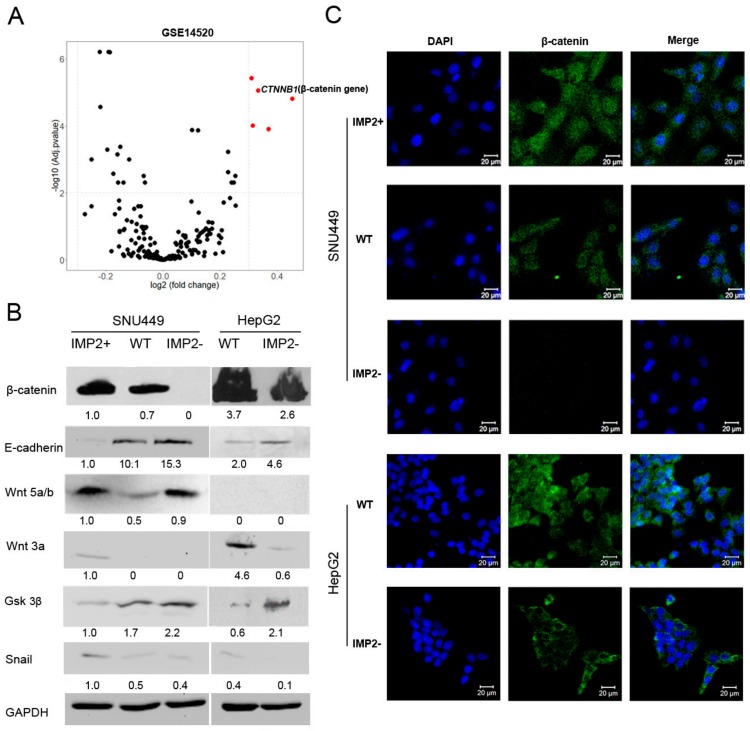
Activated Wnt/β-catenin signaling was observed in p62/IMP2 overexpression cells. (**A**) DEGs analysis with a metastasis-related gene list. The –log10 (adj. *p* value) and LogFC (log 2 conversion of the fold change) between the p62/IMP2 high expression group and p62/IMP2 low expression group was shown in a scatter diagram. (**B**) Western blotting analysis of Wnt/β-catenin signaling and EMT-related proteins. (**C**) Immunofluorescence staining of β-catenin in SNU449 and HepG2 cells. A clear nuclear expression pattern of β-catenin was shown when p62/IMP2 was expressed highly. Cell nucleuses were stained with DAPI (blue).

To better understand how the change of p62/IMP2 can regulate cell migration in liver cancer cells, we performed a differential expression gene analysis with a metastasis-related gene list (QIAGEN) between the p62/IMP2 high expression group and low expression group in GSE 14520 (Table S2). One of the top genes we selected was *CTNNB1* (based on LogFC + adj. *p* value) because we considered it as a candidate of the targets of p62/IMP2 (Figure 4A). It is involved in the regulation of Wnt signaling. Since the Wnt/β-catenin pathway plays an essential role in the process of EMT and cancer metastasis, we examined the expression of β-catenin by immunofluorescence (Figure 4C). The results indicated that the expression of β-catenin was significantly enhanced when p62/IMP2 was overexpressed and decreased in p62/IMP2 knocked-out variants (Figure 4A). HepG2 and SNU449 cell lines reported activated Wnt/β-catenin signaling since the mutations in their *CTNNB1* gene; due to this, we hypothesized that the translocation of β-catenin into the nucleus can be observed in both cell lines and their variants. However, we only observed a stronger nuclear expression pattern of β-catenin in the p62/IMP2-overexpressed cells. When p62/IMP2 was expressed in low quantities, β-catenin exhibited a more cytoplasmic expression pattern. Considering p62/IMP2 as an mRNA-binding protein, we further investigated the correlation between the *IGF2BP2* and *CTNNB1* expression in SNU449, HepG2, Hep3B, and Huh7 cell lines from the dataset GSE 97098. The results were impressive, which showed a highly positive correlation between the expression of these two genes with an R^2^ value of 0.9971 (Figure S4). The change of β-catenin expression influenced by p62/IMP2 was confirmed in western blotting analysis. In addition, we tested the expression of several key proteins that are involved in the regulation of the Wnt/β-catenin signaling pathway and EMT. The Wnt3a and Wnt5a are Wnt–protein ligands, which are necessary to activate the Wnt signaling [24]; GSK3β is one of the most important proteins that is responsible for β-catenin degradation [25]. The loss of E-cadherin is considered the fundamental event in EMT. Snail can repress the expression of E-cadherin and it is a downstream protein of the Wnt/β-catenin pathway [26]. In general, the protein expression of Wnt3a and snail presented a positive correlation with the expression of p62/IMP2 while GSK3β and E-cadherin showed a negative correlation with the expression of p62/IMP2. However, there are some unexpected results of the western blotting analysis: For example, Wnt5a/b was lost in the HepG2 cell line, overexpressed in both SNU449 IMP2-overexpressed and knockout cells. (Figure 4B). *GLUL*, the gene of glutamine synthetase, is a target of Wnt/β-catenin signaling in liver. Western blotting results revealed that the expression of p62/IMP2 has a positive correlation with the expression level of *GLUL* (Figure S5). These facts illustrated that p62/IMP2 is a regulator of the Wnt/β-catenin pathway.

### 2.5. Inhibition of β-catenin Reduces the Migration Ability of Liver Cancer Cells

**Figure 5:**
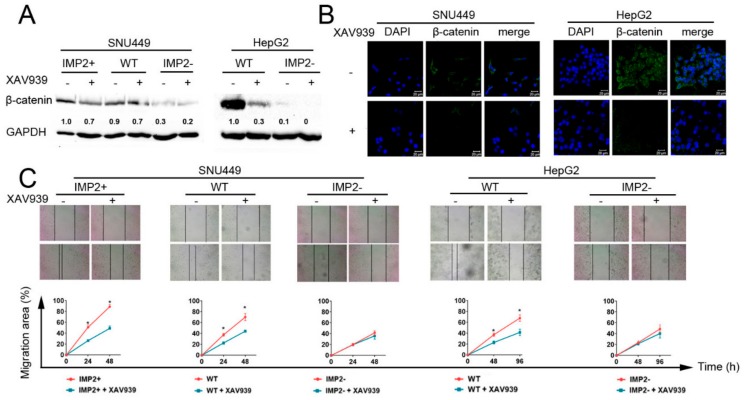
Inhibition of Wnt/β-catenin signaling can reduce migration ability in liver cancer cell lines. Liver cancer cells were cultured with 10 μM Wnt/β-catenin signaling XAV939 for 24 h before harvest. (**A**,**B**) Western blotting analysis and immunofluorescence showed β-catenin expression was significantly inhibited by 10 μM XAV939. (**C**) XAV939 repressed migration ability in p62/IMP2 overexpression and wild-type liver cancer cells. In contrast, XAV 939 did not show inhibition of migration ability in IMP2 knockout cells. A wound scratch image was taken every 24 h, the wound healing area was quantified and each point (48 h per time in the HepG2 line). * *p* < 0.05 (two-way ANOVA, *n* = 3).

We further explored whether inhibition of β-catenin expression could attenuate the migration ability of liver cancer cells. The expression of β-catenin in wild-type of HepG2 and SNU449 cells was reduced by around 30% to 60% after being cultured in 10 μM Wnt/β-catenin signaling inhibitor XAV939 medium. (Figure 5A,B). Consequently, the in vitro wound healing assay provided evidence that inhibition of β-catenin can suppress the migration ability in wild-type and p62/IMP2-overexpressed liver cancer cells, whereas no significant change was observed in p62/IMP2 knockout liver cancer cells (Figure 5C). 

### 2.6. IMP2 Can Regulate the Phosphorylation of Wnt/β-catenin Signaling

**Figure 6:**
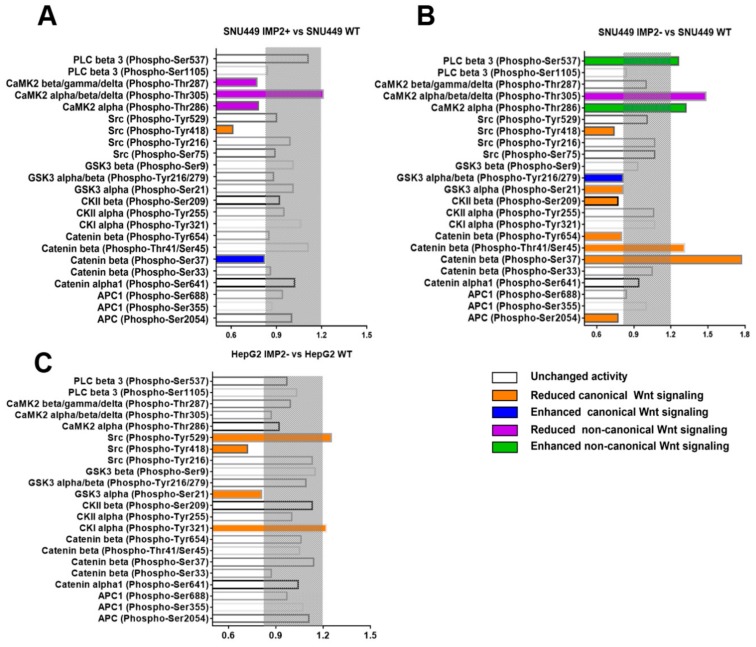
Overexpression of p62/IMP2 influenced phosphorylation in the Wnt signaling cascade in liver cancer cells. The change of the phosphorylation rate (phosphorylated protein/phosphorylated protein) between each transfected variant and their corresponding wild-type variants was tested by a phosphoproteome array, which screened the total cell lysates from each variant. The gray area was defined as the “no change area” (≥83%/≤120%). The induction/reduction of phosphorylation sites is shown with color when their bar is out of the gray area boundaries. (**A**) Fold change of indicated phosphoproteins between SNU449 IMP2+ and SNU449 WT cells. (**B**) Fold change of indicated phosphoproteins between SNU449 IMP2− and SNU449 WT cells. (**C**) Fold change of indicated phosphoproteins between HepG2 IMP2− and HepG2 WT cells.

IMP2 was reported to be involved in the post-translational modification of their targets, including phosphorylation [27]. Therefore, we performed a phosphoproteome analysis by phospho explorer antibody microarrays and then characterized and compared the phos/unphos (phosphorylation/unphosphorylation) ratio between transfected and wild-type cell lines. There are 93 well-characterized phospho-specific antibodies in the microarray, and we selected 23 Wnt-related phosphorylation sites from them. Intriguingly, the pattern of phosphorylation is diverse in each group. In general, overexpression of p62/IMP2 enhanced canonical Wnt signaling in both SNU449 and HepG2 cell lines, but the pattern of affected phosphorylation sites was different (Figure 6A,B). In SNU449, the change of p62/IMP2 mainly regulates the cytoplasmic phosphorylation sites associated with β-catenin degradation, such as β-catenin Y654, T41/S45, S37, and APC S2054. Among them, the phosphorylation ratio of β-catenin S37 was increased by 77% in p62/IMP2 knockout cells while it decreased by 19% in p62/IMP2-overexpressed cells, which promoted the degradation of β-catenin induced by GSK3b and supported our western blot results in Figure 4. In contrast, the change of p62/IMP2 in HepG2 primarily regulates the Wnt signaling receptor-associated phosphorylation site on the cell membrane (Src Y529, S75, and CKIα Y321) (Figure 6C). At the same time, in SNU449 cells, the expression of p62/IMP2 was negatively correlated with the phosphorylation site that activated non-canonical Wnt signaling (Figure 6A,B), whereas we did not find a correlation between the expression level of p62/IMP2 and the phosphorylation level of non-canonical Wnt signaling in HepG2 cells (Figure 6C).

## 3. Discussion

p62/IMP2 was first identified in 1999 as a TAA. Our previous work showed that a high frequency of its autoantibodies can be detected in sera from HCC patients, which suggests that p62/IMP2 can be used as a biomarker of HCC [14]. Nevertheless, the mechanism underlying the production and regulation of these autoantibodies remains to be explored. Previous studies revealed that most TAAs are non-mutated. They are expressed at low levels in normal tissues but highly overexpressed in tumorigenesis. Autoantibodies against these TAAs are produced when the antigenic peptides presented on human leukocyte antigen (HLA) class I molecules exceed the TCR threshold, which is required for CD4+ T cell activation [28]. In this study, we provided evidence for the hypothesis that p62/IMP2 is aberrantly overexpressed in both HCC tissues and cell lines, and our IHC results showed that the expression of p62/IMP2 reached a high level in the early stage of cancer, which is consistent with a previous study showing that autoantibodies can be detected early in HCC [29]. Considering that the overall survival time is less than 1 year for late-stage HCC patients (based on the BCLC staging system), early diagnosis is most important for improving the prognosis of patients. In summary, using autoantibodies against a panel of cancer-associated antigens, including p62/IMP2, may be a potential direction for future diagnosis of early HCC [14].

As much as 90% of cancer-related deaths can be attributed to metastasis [30]. Through the establishment of stable p62/IMP2-overexpressed and knockout cell lines, we found that aberrant highly expressed p62/IMP2 can regulate the cell migration ability of liver cancer cells via regulation of the expression of β-catenin. β-catenin is known to play a key role in the cadherin-mediated cell adhesion system. Without Wnt signaling cascade activation, the β-catenin-α-catenin complex bridges the cadherin to the actin cytoskeleton physically in the cytoplasm [31]. Once Wnt binds with Fz and LPR5/6, the Wnt/β-catenin signaling pathway activates and allows β-catenin to accumulate in the cytoplasm in order to finally translocate into the nucleus. β-catenin in p62/IMP2-overexpressed cells showed a clear nuclear expression pattern. On the contrary, β-catenin is mainly localized in the cytoplasm of HepG2 p62/IMP2 knockout, SNU449 WT, and SNU449 p62/IMP2 knockout cells. At the same time, a canonical Wnt ligand, called Wnt3a, was expressed only in p62/IMP2-overexpressed cells, which implies that Wnt/β-catenin signaling is enhanced by the overexpression of p62/IMP2. In the activated Wnt/β-catenin signaling pathway, reduction of GSK3β reduces the phosphorylation of Snail, thereby enhancing its stability [26], inhibiting the expression of E-cadherin, and ultimately, inducing the occurrence of EMT. 

Mutations of HCC in the Wnt/β-catenin pathway frequently occur in HCC patients (~30% in *CTNNB1* and ~10% in *AXIN1*) as well as multiple liver cancer cell lines. The mutations also occur in liver cancer cell lines. In our study, SNU449 and HepG2 cells contain mutations in *CTNNB1*; Hep3B and SNU449 cells have mutations in *AXIN1*. In general, the mutations in *CTNNB1* and *AXIN1* can prevent the degradation of β-catenin and facilitate its accumulation, finally activating canonical Wnt signaling. However, the Huh7 cell line, which contains wild-type β-catenin and Axin-1, has higher TCF activity than the Hep3B and SNU449 cell lines [24]. Our study may reveal one of the reasons for this puzzling result: Huh7 cells have the highest IMP2 mRNA expression in the cell lines we used. Because of the high correlation between β-catenin mRNA and IMP2 mRNA expression, Huh7 cells also expressed the highest β-catenin mRNA, and that may trigger the accumulation of β-catenin (Figure S4). Therefore, the regulation of mRNA expression may be another important way to activate Wnt signaling besides mutations. 

Surprisingly, although the expression of β-catenin was intensely repressed and E-cadherin was enhanced in SNU449 p62/IMP2 knockout cells, the cells did not express a significantly changed colony formation ability compared with SNU449 wild-type cells. The Wnt signaling pathway contains the canonical pathway and noncanonical pathway and both can be activated when a Wnt protein binds to a Frizzled family receptor [21,32]. The difference between them is the noncanonical pathway, which is β-catenin independent and it can antagonize canonical Wnt signaling in HCC [24]. A study showed that Wnt receptor, Frizzled 2, and its ligand, Wnt5a/b, were found to be elevated in various cancers that also promote proliferation and cell migration ability [21]. Liver cancer cell lines express Wnt ligands diversely [24]. Briefly, well-differentiated cell lines, such as HepG2, mainly express Wnt3a, whereas poorly differentiated cell lines like SNU449 mainly secrete Wnt5a/b [21]. Due to this, we examined the noncanonical Wnt signaling pathway. In the scenario of the sharp downregulation of β-catenin in SNU449 p62/IMP2 knockout cells, Wnt5a/b may be oppositely upregulated and thus trigger the process of proliferation. p62/IMP2 is the only IMP family member that was reported to exist in adult liver tissues [9]. Our study suggests that the traces of p62/IMP2 expression are important to maintain normal function in hepatocytes.

In the Wnt pathway, protein activity, stability, and binding properties were regulated by the phosphorylation status at multiple steps [33]. In this study, we showed that manipulation of the expression of p62/IMP2 triggers a series of changes in the phosphorylation of various Wnt pathway proteins. In the absence of a Wnt stimulus, free β-catenin in the cytoplasm will be degraded by a “destruction complex”, including APC, GSK3, Axin, and CK1 [25]. In SNU449 p62/IMP2 knockout cells, CK1α-mediated phosphorylation of ser45 on β-catenin was elevated, which allowed β-catenin to enter the “destruction complex” and specifically interact with Axin [34]. Subsequently, with the dramatic increase of GSK3-mediated phosphorylation of Ser-37 and Thr-41, β-catenin binds with APC, stays in this complex, and is eventually degraded [32]. Simultaneously, the affinity for cadherins is strengthened with the reduction of Tyr 654 phosphorylation on β-catenin [35]. Decreased phosphorylation of APC at ser 2054 can facilitate the APC to be transported to the nucleus [36]. Besides that, the AKT-mediated phosphorylation of GSK3α at ser 21 significantly inhibits the activity of itself [37] (Figure 6B). In summary, all of these changes in phosphorylation strengthen the stability of β-catenin and enhance the Wnt/β-catenin signaling.

In HepG2 cells, the phosphorylation regulated by p62/IMP2 showed a different pattern. Phosphorylation of Tyr 418 of Src was inhibited after knocking out p62/IMP2. Activated proto-oncogene tyrosine kinase Src can enhance the cap-dependent translation, and thus elevate the expression of β-catenin accumulation and its transcriptional activity. Tyr 418 and Tyr 529 are two major phosphorylation sites on Src, and Tyr 418 can be autophosphorylated, and then displaced from binding pockets to allow substrate access. On the contrary, phosphorylated Tyr 529 can let the tyrosine group interact with the SH2 domain and inactive Src [38]. In addition, reduced ser 21 on GSK3α also downregulated Wnt signaling. We did not observe the phosphorylation change on β-catenin, which may explain why the downregulation of β-catenin is less in p62/IMP2 knockout cells of HepG2 cells than SNU449 cells (Figure 6C). 

Wnt5a can induce CaMKII to weaken the protein stability of a co-repressor of Notch signaling and the silencing mediator of retinoic acid and thyroid hormone receptor (SMRT). Phosphorylated Tyr 287 of CaMKII (Tyr 286 in the α isoform) plays an important role in it by a more than 1000-fold increase in the binding affinity between CaM and CaMKII, and preventing auto-inhibition when CaM dissociates from CaMKII due to the [Ca2+] i decrease [39]. Moreover, CaMKII can phosphorylate Ser 537 of PLCβ3 and thus enhance its basal activity [40]. Our phosphoproteome analysis data supported that loss of p62/IMP2 augments Wnt5-mediated non-canonical Wnt signaling, which may explain why the colony formation ability is not reduced in SNU449 p62/IMP2 knockout cells. Nevertheless, the IMP2 overexpressed SNU449 showed both increased Wnt3a and Wnt5a, which suggests the regulation between canonical and noncanonical Wnt signaling by p62/IMP2 has a partial reliance and remains to be explored in future work. Our results showed that p62/IMP2 regulates the Wnt/beta-catenin pathway through multiple pathways: Regulation of mRNA expression, thus affecting multiple upstream and downstream genes, and changes of phosphorylation levels. For instance, even we did not observe a lot of alternation in the phosphorylation sites of the Wnt/beta-catenin pathway proteins in p62/IMP2-overexpressed cells, and p62/IMP2 still can enhance Wnt/beta-catenin via regulation of the mRNA expression of related genes.

In the differential gene expression analysis, we found that 900 genes were regulated by the change of expression; in addition, their encoded proteins were mainly distributed in the cell membrane and cytoplasm, rather than in the nucleus. Another study showed that 3% of the HEK293 cell transcriptome will be expressed in IMP1 messenger ribonucleoprotein (mRNP) particles, which implies that there may be over 1000 targets of IMP1 [41]. Given that IMP family members share a highly conserved structure, with 60% to 80% identity amino acid sequences [42], there may be many unknown IMP2 targets in these differentially expressed genes, which can help regulate complex pathways, their phosphorylation, and improve the migration ability of cancer cells. In the future, to better understand how p62/IMP2 regulate Wnt/beta-catenin, we can perform the TCF reporter assay and test more targets in liver, such as OAT, LECT2, etc. Moreover, we plan to conduct a RIP assay with an anti-p62/IMP2 antibody to reveal all the mRNAs that can bind to p62/IMP2. Therefore, we can identify whether β-catenin mRNA is a direct target of p62/IMP2, and if Wnt/beta-catenin can be regulated by the multiple p62/IMP2 target mRNAs. 

## 4. Materials and Methods

### 4.1. Data Pre-processing

All the original data were downloaded from GEO for further pre-processing. R software (version 3.4.3) was used to read and analyze data. The bioconductor “affy” package was used to adjust the background and normalize the original microarray data. Probe id were annotated to gene id accordingly. For genes with multiple detecting probes, mean gene expression values were used in the subsequent analyses. 

### 4.2. Differential Expression Analysis

The gene expression profile from HCC patients or HCC tissues or HCC cell lines were included. Due to the nature of the different types of sample sources, they were analyzed separately. All samples were separated into two groups, a high expression group and a low expression group, by using the median expression value of the *p62*/*IMP2* gene. The bioconductor package “limma” was used to compare differentially expressed genes (DEGs) in the high and low p62/IMP2 expression groups. Genes with a log2 fold change greater than 1.5 and adjusted *p* value lower than 0.01 were considered significantly different.

### 4.3. Gene Ontology (GO) and KEGG Annotation of DEGs

The R Bioconductor package “clusterProfiler” was used to carry out GO annotations for the DEGs. 

### 4.4. Cell Lines and Cell Culture

Human liver cancer cells SNU449 and HepG2 were purchased from ATCC and cultured in RPMI 1640 (GIBCO, Life Technologies, Grand Island, NY, USA). The medium was supplemented with 10% FBS and 100 units/mL penicillin plus 100 μg/mL streptomycin (thermo Scientific, Waltham, MA, USA) at 37 °C and 5% CO_2_. When cells were cultured with the Wnt/β-catenin inhibitor, 10 μM of XAV-939 was added into the media at 80% cell confluence. The cells were collected after 24 h of growth. 

### 4.5. Transfection

To overexpress p62/IMP2, SNU449 cells were transfected using Lipofectamine 2000 (Life Technologies, Grand Island, NY, USA) in 6-well plates following the manufacturers’ instruction. In total, 800 ng/mL G418 was used to select the transfected cells and to obtain stably transfected clones. The clones were picked with cloning cylinders (Corning, Tewksbury, MA, USA) and expanded in 24-well plates.

To knock out p62/IMP2, co-transfected IMP2 HDR Plasmid (h) and IMP2 CRISPR/Cas9 KO Plasmid (h) (Santa Cruz Biotechnology, Dallas, TX, USA) were used with Lipofectamine 3000 (Life Technologies, Grand Island, NY, USA). Puromycin (1 mg/mL) was used to select stable p62/IMP2 knockout variants. In total, 10 clones for each variant were obtained, and they were expanded and tested by western blotting.

### 4.6. Immunohistochemistry

Immunohistochemistry was performed on a commercially available liver cancer tissue array (BC03116a, US Biomax, Inc., Derwood, MD, USA). Briefly, after deparaffinization with xylene and rehydration with ethanol, antigen retrieval was performed by microwave heating methods. First, 2 to 3 drops of avidin block solution were applied to the slide to block endogenous biotin activity. After washing, 200 μL of p62/IMP2 antibody (ABCAM, Cambridge, MA, USA) with 1:100 dilution was applied to the cover of every slide and then incubated overnight at 4 °C. The slide was then incubated with polyvalent biotinylated-linked goat-anti-rabbit secondary antibody for 1 h at room temperature. 3′-Diaminobenzidine (DAB) was used for detection. Lastly, the slide was counterstained with hematoxylin, dehydrated with ethanol, stabilized with xylene, and then processed for imaging. 

### 4.7. Proliferation Assay

Cells were seeded at 5 × 10^3^ cells/well in 96-well plates and grown for 1 to 5 days. The cell proliferation was determined by a sulforhodamine (SRB) assay. Briefly, cells were fixed with 10% (*w/v*) trichloroacetic acid (TCA) at 4 °C for 1 h, rinsed 5 times with water, and air dried. The cells were then fixed with 100 μL 0.4% (*w/v*) SRB in acetic acid for 15 min. After washing, the unbound dye was removed by washing five times with 1% acetic acid and the plate was air-dried. The binding stain was dissolved by 150 μL 10 Mm Tris base for each well. Colorimetric readings were performed in a microplate reader at 515 nm.

### 4.8. Transwell Migration Assay

Cells were detached from the culture plate with 0.25% Trypsin-EDTA solution. After centrifugation, the cells were resuspended in serum-free medium, and 100 μL of the cell suspension was added onto the filter of a transwell chamber. The cells were cultured for 16 h after carefully adding 600 μL of 10% FBS medium in 24-well plate lower chambers. Cells were fixed by 3.7% formaldehyde in PBS and then permeabilized by 100% methanol for 20 min at room temperature. Cells were finally stained with 0.1% crystal violet for 15 min and counted under a microscope. Between each step, cells were washed twice with PBS.

### 4.9. Transwell Invasion Assay

The Matrigel (Corning, Tewksbury, MA, USA) was diluted at 1:30 on ice with serum-free RPMI 1640 medium, 100 μL of diluted Matrigel was added into each transwell chamber, and incubated at 37 °C overnight. The following steps were exactly same as the method stated in Section 4.8. for the transwell migration assay.

### 4.10. Plate Clonogenic Assay

Cells were seeded into 6-well plates at 500 cells/well and cultured for 2 weeks with 10% FBS. The colonies were washed with PBS, fixed with 100% methanol for 15 min, and stained with 0.1 % crystal violet for 20 min. Colonies with more than 50 cells were counted manually. 

### 4.11. Wound Healing Assay

Cells were cultured in 6-well plates and cultured in RPMI 1640 medium with 2% FBS. Wound scratches were made to the all monolayer cells with pipetting tips. The photographs of the wound scratches were taken every 24 h. The cell migration level on the scratch was quantified using Image J software (https://imagej.nih.gov/ij/).

### 4.12. Western Blot Analysis 

Cells for western blotting were cultured in RPMI 1640 medium with 10% FBS for 24 h. Protein expression was detected with rabbit antibodies: p62/IMP2, β-catenin, Wnt3a, Wnt 5a/b, GSK3β, Snail, N-cadherin, and GAPDH (Cell Signaling Technology, Danvers, MA, USA); and mouse antibodies: E-cadherin (Cell Signaling Technology, Danvers, MA, USA). Primary antibodies were detected with goat anti-mouse IgG (HPR conjugate) or anti-rabbit IgG (HPR conjugate). The membranes were finally scanned by Invitrogen iBright Imaging System (Thermo Fisher Scientific, Waltham, MA, USA).

### 4.13. Immunofluorescence

Cells were seeded into 8-chamber culture slides and grown for 24 h fixed with 100% methanol and 100% acetone at −20 °C. The cells were subsequently treated with β-catenin antibodies (Cell Signaling Technology, Danvers, MA, USA) and incubated with Alexa Flour 488 conjugate secondary antibodies. Mounting medium containing DAPI was then added. Confocal fluorescence images were acquired with a laser scanning microscope (LSM 700; Zeiss, New York, NY, USA). 

### 4.14. Phosphoproteome Assay

The Wnt Pathway Phospho Antibody Array (PNT227) was conducted by Full Moon BioSystem Inc, which contains 227 site-specific and phospho-specific antibodies. SNU449 cells, HepG2 cells, and their transfected variants were harvested as whole-cell lysates and transferred to Full Moon Biosystem Inc in dry ice. Further, proteins were purified, labeled with biotin, and placed on the blocked microarray with coupling solution. Then, 0.1% Cy3-Streptavidin solution was used to detect conjugation-labeled proteins. The phosphorylation degree of a site was represented by the ratio of phosphorylation to unphosphorylation.

## 5. Conclusions

In summary, our study revealed that p62/IMP2 is overexpressed in human HCC tissues and cell lines, thereby augmenting cell migration ability through activation of the canonical Wnt signaling pathway. Furthermore, our differential expression analysis and GO analysis indicated that abnormally overexpressed p62/IMP2 in HCC may have more mRNA targets and can regulate the cancer progression through a variety of pathways, resulting in more malignant HCC phenotypes.

## Figures and Tables

**Table 1:** Expression of p62/IMP2 in HCC tissues and adjacent normal tissues tested by IHC.

Type of Tissues	IHC score			
	Sample Quantity	Color	Area	Final Score
Tumor tissues	40	2.823 *	3.68	10.30 *
Stage I tumor tissues	4	2.50 *	3.75	9.00 *
Stage II tumor tissues	13	2.85 *	3.54	10.15 *
Stage III tumor tissues	23	2.87 *	3.74	10.61 *
Normal tissues	30	1.5	3.67	5.23

* *p* < 0.05, compared with the normal group. (one-way ANOVA). The staining of p62/IMP2 was evaluated by a four-level scoring system for color and area. The final score is the product of the color and area score. Here, we show the average number of samples.

## Supplementary Materials

The following are available online at https://www.mdpi.com/2072-6694/12/1/7/s1, Figure S1: The FunRich enrichment analysis of Biological pathway and Cellular component for 900 DEGs. The enrichment analyses were performed with FunRich 3.1.3 software, Figure S2: Gene Interaction analysis of metastasis related DEGs. The selected metastasis related DEGs are shown as red and their directly interacted gene are shown as green, Figure S3: The transwell invasion assay in p62/IMP2 variants from two liver cancer cell lines. * *p* < 0.05, ** *p* < 0.01, transfected cells compared with wildtype cells, Figure S4: The correlation between the expression of *CTNNB1* and *IMP2* in human liver cancer cell lines. The data was obtained from GSE 97098 datasets, Figure S5: The expression of *GLUL* in p62/IMP2 variants from two liver cancer cell lines was shown by western blotting analysis, Figure S6: Original western blots with molecular weight markers, Table S1: Differentially expressed gene between *IGF2BP2* high expression group and low expression group, Table S2: Differential expression analysis between *IGF2BP2* high expression group and low expression group with a metastasis related gene list.
