# Supplementary material for: Overexpression of p62/IMP2 can Promote Cell Migration in Hepatocellular Carcinoma via Activation of the Wnt/β-Catenin Pathway
Source: Cancers (Basel). 2019 Dec 18;12(1):7. doi: 10.3390/cancers12010007 (PMC7017416; doi:10.3390/cancers12010007)
Supplement: Supplementary file 1 [file cancers-12-00007-s001.zip › cancers-643591-supplementary materials/cancers-643591-supplementary (Figures).pdf]

Article

Overexpression of p62/IMP2 can Promote Cell Migration in Hepatocellular Carcinoma via Activation of the Wnt/ β-Catenin Pathway

Mengtao Xing, Pei Li, Xiao Wang, Jitian Li, Jianxiang Shi, Jiejie Qin, Xiaojun Zhang, Yangcheng MA, Giulio Francia* and Jian-Ying Zhang *

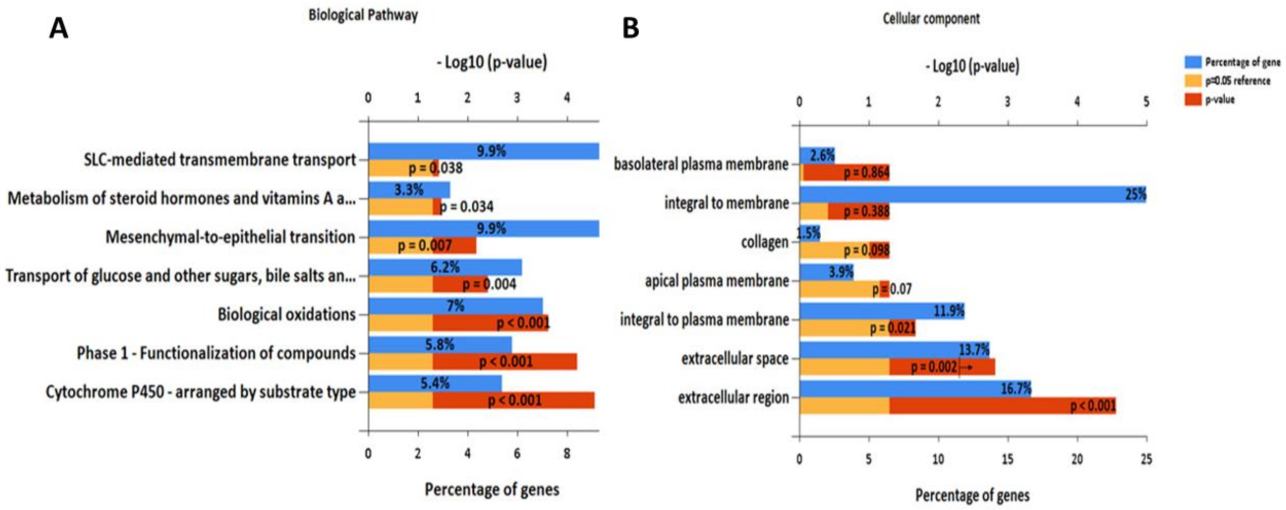

Figure S1. The FunRich enrichment analysis of the biological pathway and cellular component for 900 DEGs. The enrichment analyses were performed with FunRich 3.1.3 software.

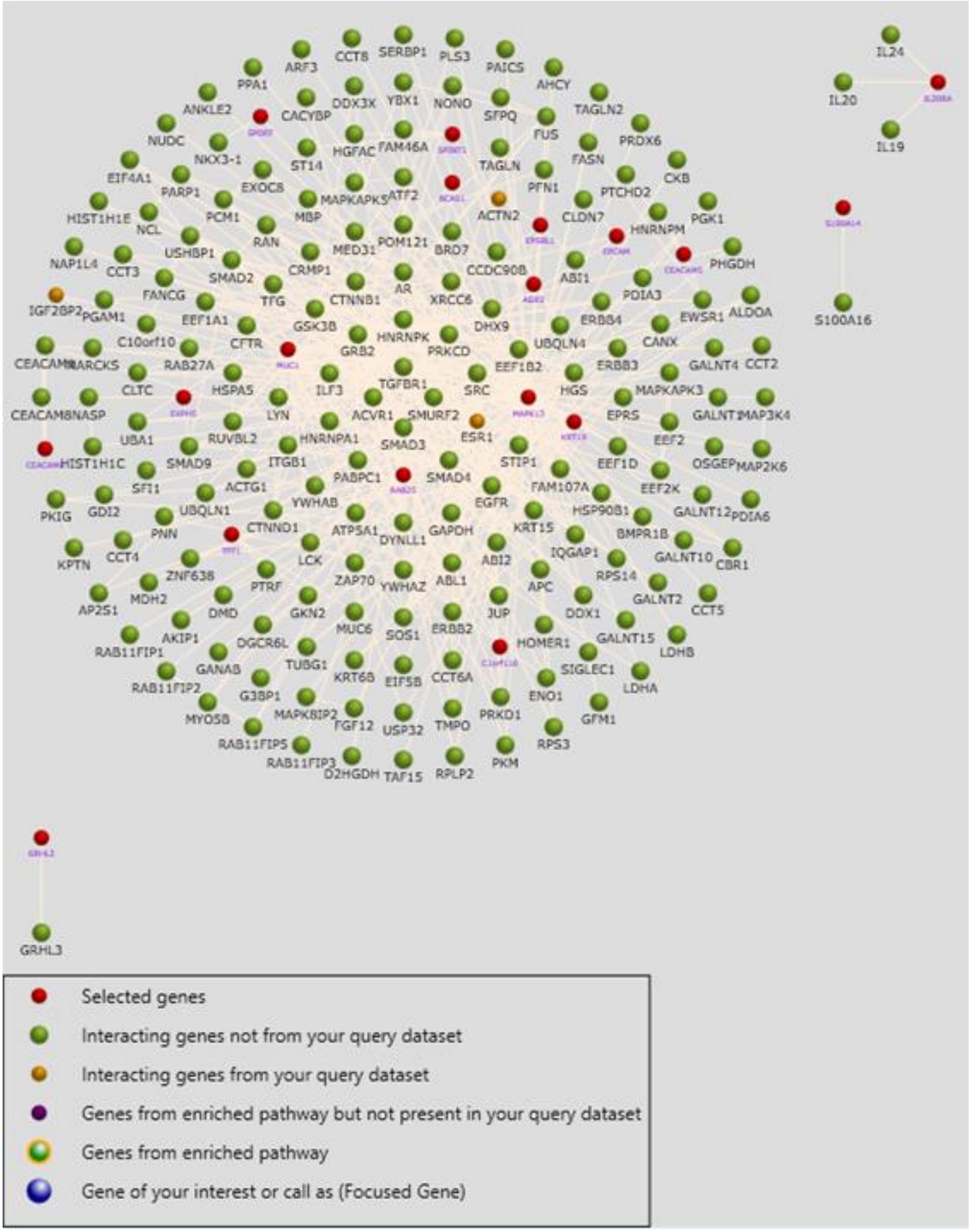

Figure S2. Gene interaction analysis of metastasis-related DEGs. The selected metastasis-related DEGs are shown in red and their directly interacted gene is shown as green.

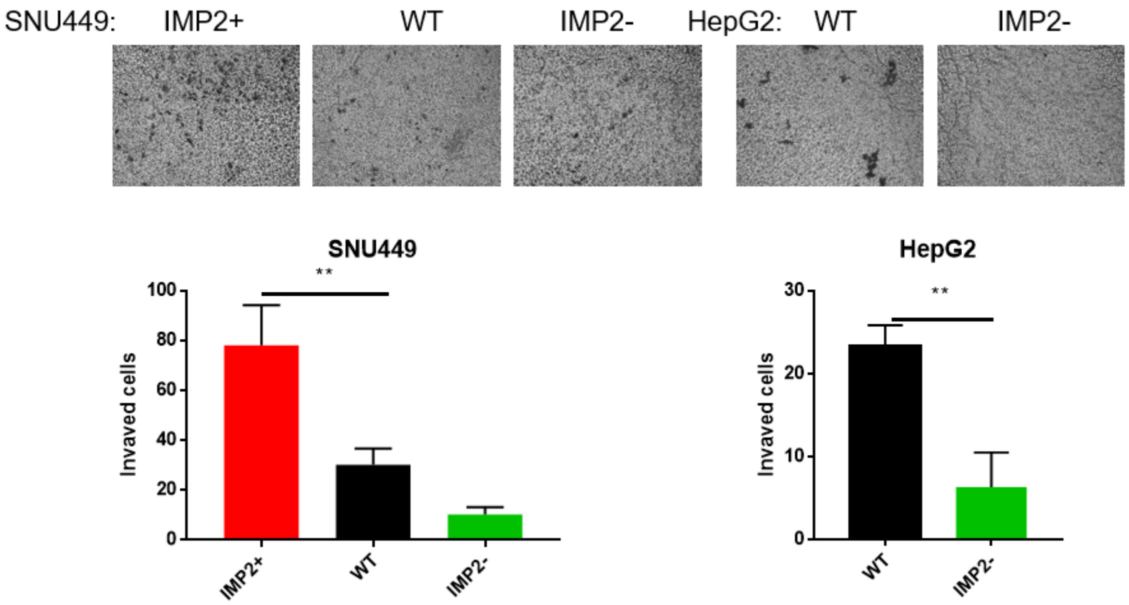

Figure S3. The transwell invasion assay in p62/IMP2 variants from two liver cancer cell lines. *p < 0.05, ** p < 0.01, transfected cells compared with wild-type cells.

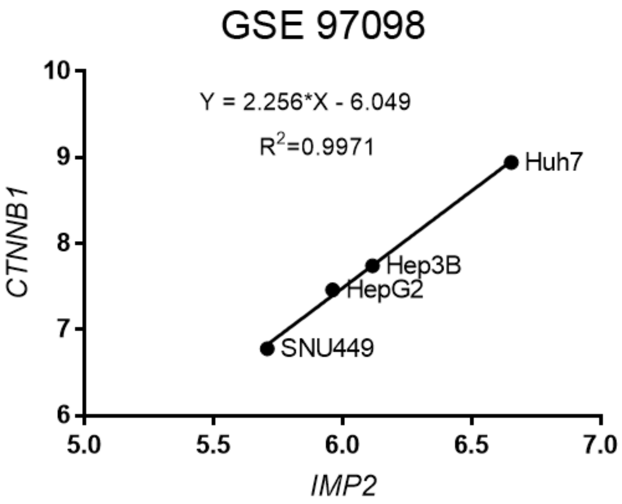

Figure S4. The correlation between the expression of *CTNNB1* and *IMP2* in human liver cancer cell lines. The data were obtained from GSE 97098 datasets.

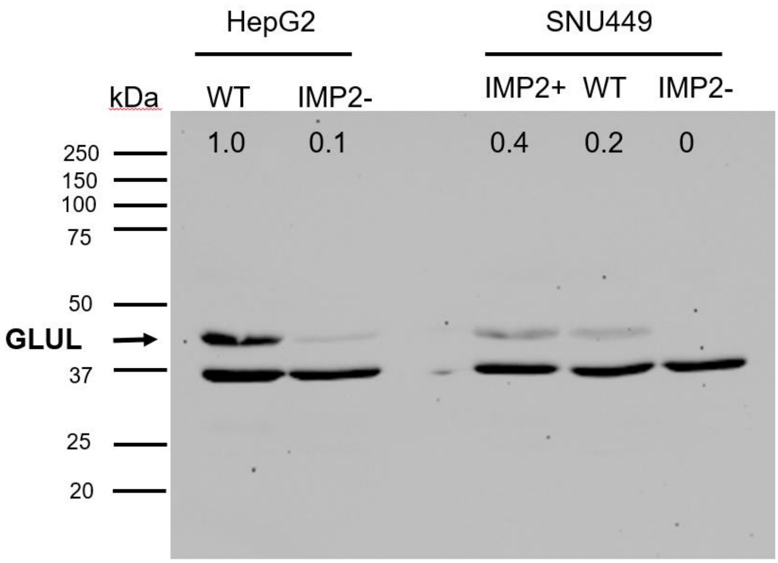

Figure S5. The expression of GLUL in p62/IMP2 variants from two liver cancer cell lines as shown by western blotting analysis.

Figure 1C

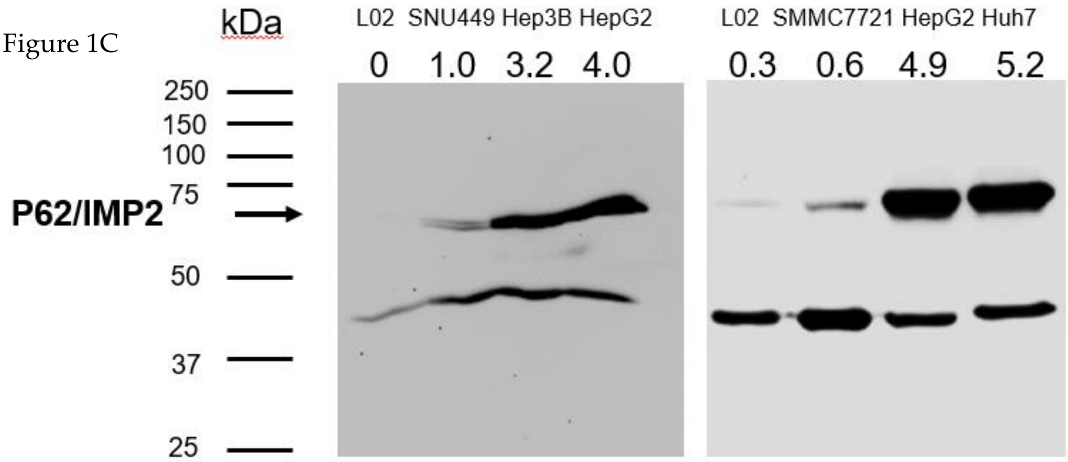

Figure 1D

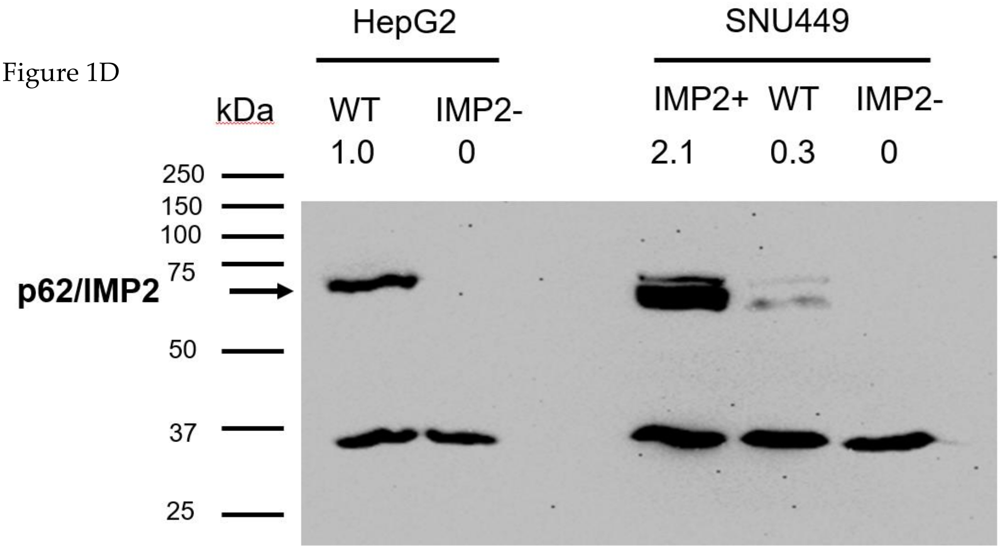

Figure 4B

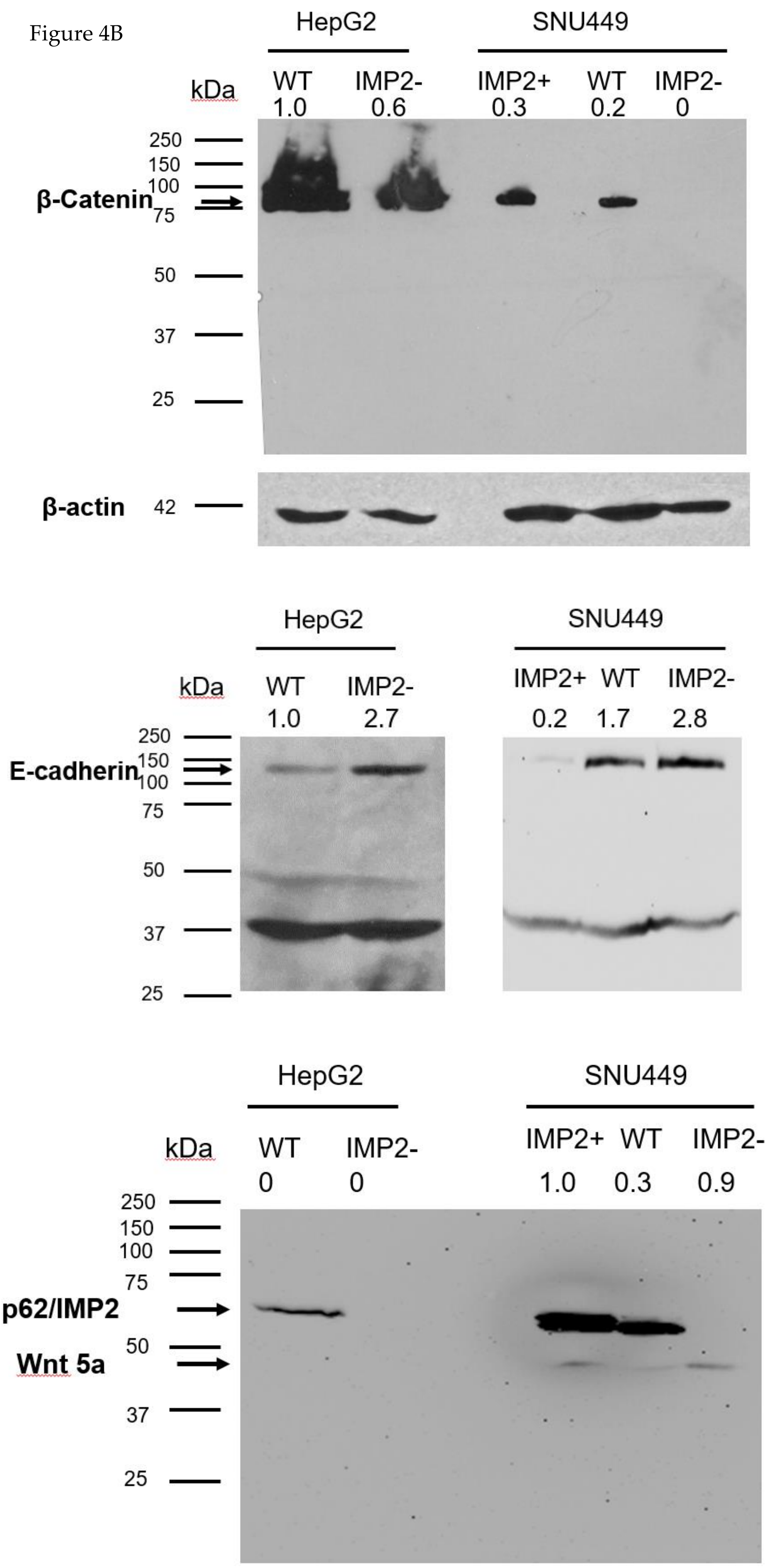
HepG2
SNU449
kDa
WT 1.0
IMP2- 0.6
IMP2+ 0.3
WT 0.2
IMP2- 0
250
150
100
75
50
37
25
β-Catenin
β-actin
42
HepG2
SNU449
kDa
WT 1.0
IMP2- 2.7
IMP2+ 0.2
WT 1.7
IMP2- 2.8
250
150
100
75
50
37
25
E-cadherin
HepG2
SNU449
kDa
WT 0
IMP2- 0
IMP2+ 1.0
WT 0.3
IMP2- 0.9
250
150
100
75
50
37
25
p62/IMP2
Wnt 5a

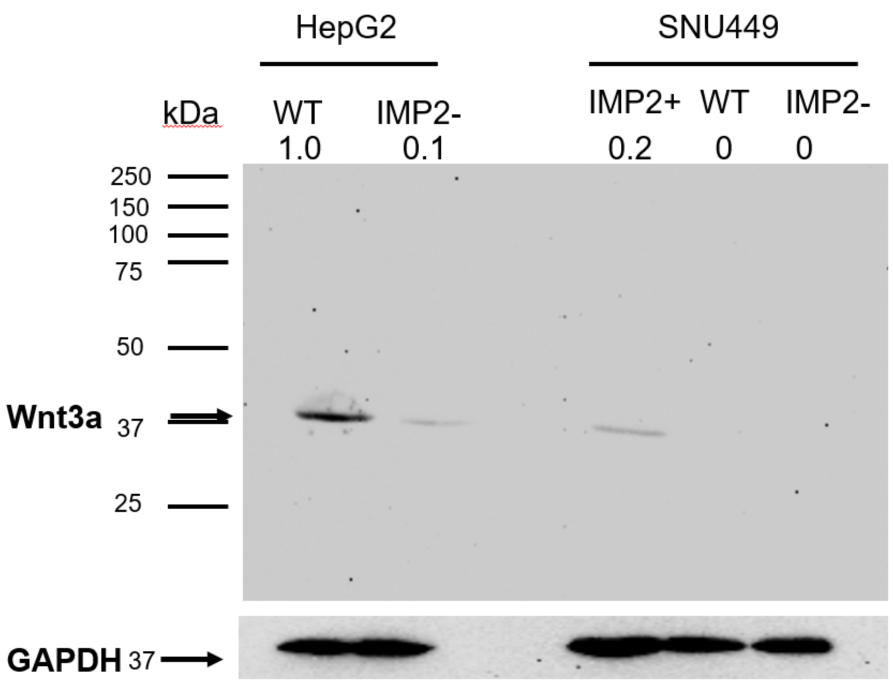
HepG2
SNU449
kDa
WT
IMP2-
IMP2+
WT
IMP2-
1.0
0.1
0.2
0
0
250
150
100
75
50
Wnt3a
37
25
GAPDH
37

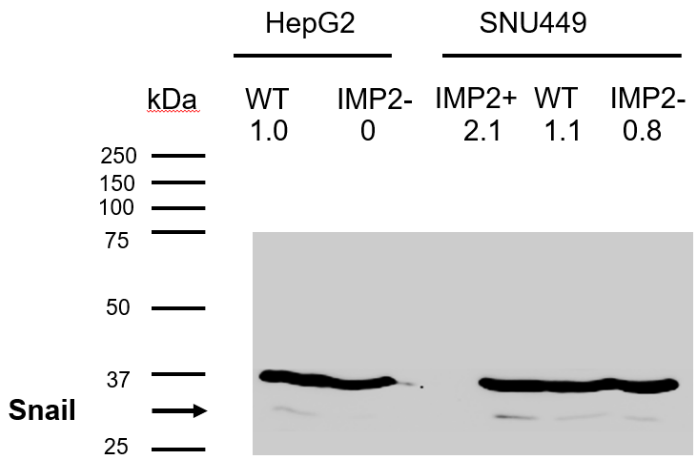
HepG2
SNU449
kDa
WT
IMP2-
IMP2+
WT
IMP2-
1.0
0
2.1
1.1
0.8
250
150
100
75
50
37
Snail
25

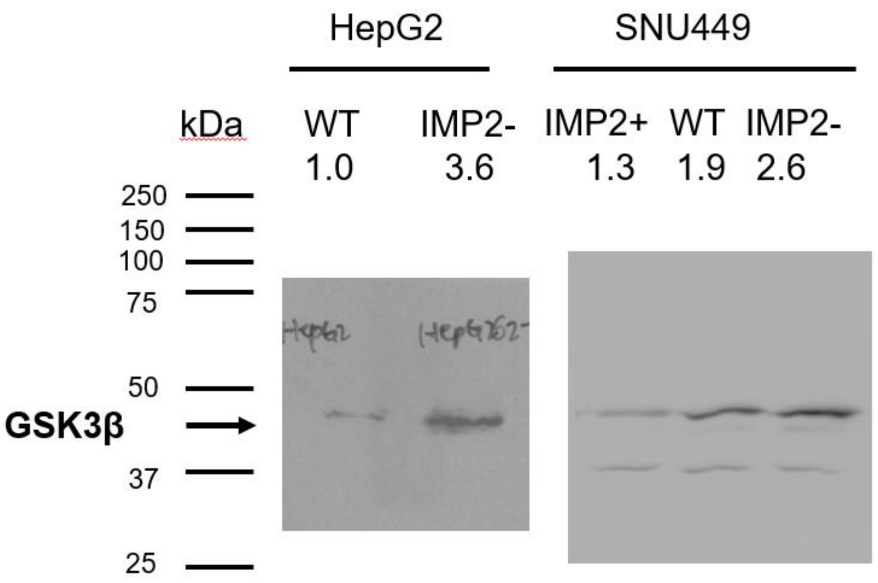
HepG2
SNU449
kDa
WT
IMP2-
IMP2+
WT
IMP2-
1.0
3.6
1.3
1.9
2.6
250
150
100
75
50
GSK3β
37
25

Figure 5A

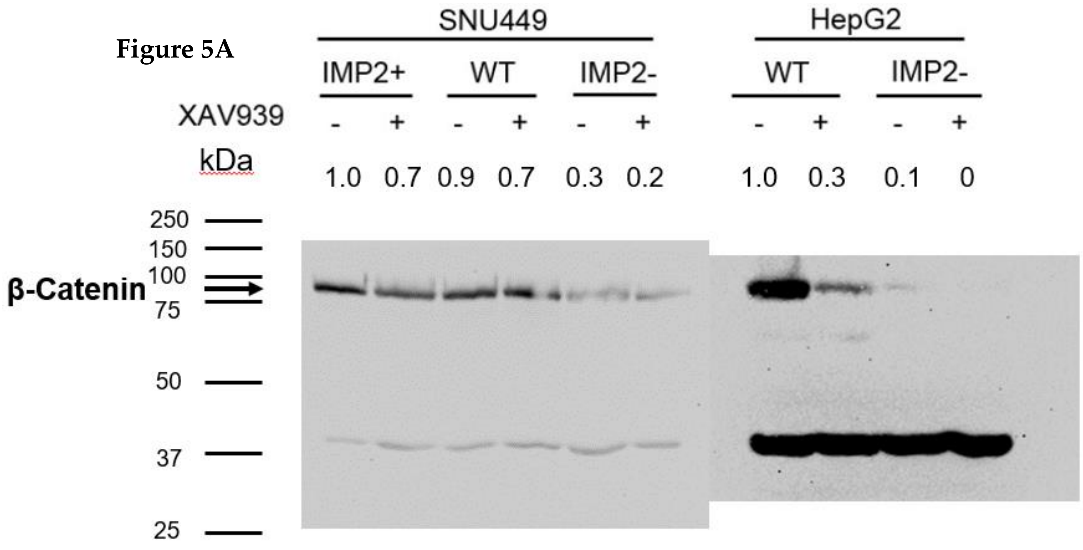

Figure S6. Original western blots with molecular weight markers.

© 2019 by the authors. Licensee MDPI, Basel, Switzerland. This article is an open access article distributed under the terms and conditions of the Creative Commons Attribution (CC BY) license (http://creativecommons.org/licenses/by/4.0/).
